# Addressing Sensor Data Heterogeneity and Sample Imbalance: A Transformer-Based Approach for Battery Degradation Prediction in Electric Vehicles

**DOI:** 10.3390/s25113564

**Published:** 2025-06-05

**Authors:** Bi Wu, Shi Qiu, Wenhe Liu

**Affiliations:** 1 Anderson School of Management, University of California, Los Angeles, CA 90095, USA; shiqiu7@g.ucla.edu; 2 School of Computer Science, Carnegie Mellon University, Pittsburgh, PA 15213, USA

**Keywords:** degradation prediction, electric vehicles, RUL estimation, data heterogeneity, sample imbalance, battery sensors

## Abstract

Battery health monitoring and remaining useful life (RUL) estimation for electric vehicles face two significant challenges: battery data heterogeneity and sample imbalance. This study presents a novel approach based on Transformer architecture to specifically address these issues. We utilized the NASA lithium-ion battery cycling dataset, which contains charge-discharge and impedance measurement data under various temperature conditions. To tackle data heterogeneity, we developed a multimodal feature fusion strategy that effectively integrates battery sensor data from different sources and formats, including time-series charge-discharge sensor data and spectral impedance sensor measurements. To mitigate sample imbalance, we implemented an adaptive resampling technique and hierarchical attention mechanism, enhancing the model’s ability to recognize rare degradation patterns. Our Transformer-based model captures long-term dependencies in the battery degradation process through its self-attention mechanism. Experimental results demonstrate that the proposed solution significantly improves battery degradation prediction accuracy, achieving a 21.3% increase in accuracy when processing heterogeneous data and a 24.5% improvement in prediction capability for imbalanced samples compared to traditional methods. Additionally, through case studies, we validate the applicability of this method in actual electric vehicle battery management systems, providing reliable data support for battery preventive maintenance and replacement decisions. The findings have important implications for enhancing the reliability and economic efficiency of electric vehicle battery management systems.

## 1. Introduction

Electric vehicles (EVs) have gained significant market share in recent years as a sustainable alternative to conventional fossil fuel-powered vehicles. A critical component determining the performance, reliability, and cost-effectiveness of EVs is the lithium-ion battery system [1]. The accurate prediction of battery degradation and remaining useful life (RUL) is essential for optimizing battery management systems, reducing maintenance costs, and enhancing user confidence in EV technology [2]. Battery degradation is a complex process influenced by multiple factors, including charging-discharging cycles, temperature variations, and usage patterns [3]. The degradation mechanisms operate across different time scales and manifest in various measurable parameters, creating a multidimensional problem that requires sophisticated analysis techniques. Traditional model-based approaches for battery health monitoring typically rely on electrochemical models or equivalent circuit models, which often struggle to capture the nonlinear degradation processes under diverse operating conditions [4]. While these physics-based models provide valuable insights into degradation mechanisms, their computational complexity and parameter sensitivity limit their application in real-time battery management systems. In recent years, data-driven approaches, particularly deep learning methods, have demonstrated promising results in battery degradation prediction [5]. These methods can automatically extract features from raw data and learn complex patterns without requiring detailed knowledge of underlying electrochemical processes.

However, despite these advances, significant challenges remain in achieving robust and accurate predictions across different sensor configurations, operational conditions and battery types. Two significant challenges persist in the application of deep learning for EV battery health monitoring. First, battery sensor data heterogeneity arises from various sensing modalities and formats of battery operational data, including time-series sensor data (voltage sensors, current sensors, temperature sensors) and frequency-domain sensor data (electrochemical impedance spectroscopy sensors) [6]. These heterogeneous data types contain complementary information about battery health but are difficult to integrate effectively within a unified prediction framework. Current approaches typically focus on either time-domain or frequency-domain data, failing to leverage the rich information contained in their combination. Furthermore, different sampling rates, measurement scales, and noise levels across data types complicate the fusion process and often lead to information loss or distortion. Second, sample imbalance is prevalent in battery degradation datasets, where normal operating data significantly outnumbers degradation data, especially for severe degradation patterns [7]. This imbalance can bias machine learning models toward majority classes, leading to poor prediction performance for critical degradation scenarios. Additionally, the scarcity of end-of-life data creates challenges in developing robust prediction models for late-stage degradation. Conventional resampling techniques often fail to address this issue effectively due to the temporal dependencies in battery data, where simple oversampling can introduce unrealistic patterns while undersampling might discard valuable information about degradation progression.

Transformer models, originally developed for natural language processing tasks [8], have recently shown potential in time-series prediction due to their ability to capture long-range dependencies through the self-attention mechanism. Their capacity to process sequences of arbitrary length while maintaining contextual information makes them promising candidates for battery degradation modeling [9]. The self-attention mechanism can dynamically focus on relevant parts of the input sequence, potentially addressing both heterogeneity and imbalance issues by learning context-dependent representations of battery health indicators.

This paper presents a novel Transformer-based approach specifically designed to address the dual challenges of data heterogeneity and sample imbalance in EV battery degradation prediction. Our approach leverages the inherent strengths of Transformer architectures while incorporating domain-specific modifications to enhance their effectiveness for battery health monitoring. The proposed method processes heterogeneous battery data through parallel Transformer encoders with shared attention mechanisms, enabling cross-modal information exchange while preserving the unique characteristics of each data type. For addressing sample imbalance, we implement a novel temporal attention mechanism that adaptively weights different degradation phases, ensuring adequate representation of rare but critical degradation patterns in the model training process.

1. A multimodal feature fusion strategy that effectively integrates heterogeneous battery data from different sources and formats, enabling comprehensive health state assessment.
2. An adaptive resampling technique combined with a hierarchical attention mechanism to mitigate sample imbalance and enhance the model’s sensitivity to rare degradation patterns.
3. A modified Transformer architecture that captures both short-term dynamics and long-term dependencies in battery degradation processes, providing accurate predictions across different operational phases.
4. Extensive evaluation using the NASA battery dataset, demonstrating significant improvements in prediction accuracy, especially for heterogeneous data and imbalanced samples.

The remainder is organized as follows: Section 2 reviews related work in battery degradation prediction, battery health monitoring, and time series prediction. Section 3 introduces some prelinimary contents, like the problem formulation, transformer architecture. Section 4 describes the proposed methodology, including model architecture, each component details, and training strategies. Section 5 presents the experimental setup and results. Finally, Section 6 concludes the paper and suggests directions for future research.

## 2. Related Works

### 2.1. Battery Degradation Prediction Methods

Battery degradation prediction has evolved from physics-based models [10] to data-driven approaches. Physics-based models provide mechanistic insights but are computationally intensive and require detailed material knowledge. Equivalent circuit models [11] offer simplified representations but struggle with complex nonlinear degradation processes. Data-driven approaches have gained popularity due to their ability to learn from historical data without detailed knowledge of underlying physics. Traditional machine learning methods, including support vector machines [12] and Gaussian process regression [5], have shown promising results but typically rely on handcrafted features and struggle with temporal dependencies in battery data. Two significant challenges persist in this domain: data heterogeneity and sample imbalance. Data heterogeneity arises from various sources and formats of battery operational data, making it difficult to integrate information from multiple sensors effectively [13]. Sample imbalance occurs because normal operating data significantly outnumbers degradation data, particularly for end-of-life scenarios, biasing models toward normal conditions [14].

### 2.2. Deep Learning for Battery Health Monitoring

Deep learning approaches offer superior feature extraction capabilities for battery health monitoring. LSTM networks have been widely applied for battery RUL prediction due to their ability to capture temporal dependencies [15]. Li et al. [16] enhanced LSTM-based prediction by incorporating attention mechanisms to focus on critical temporal patterns. CNN-LSTM hybrid models [17] leverage CNN’s feature extraction capability and LSTM’s temporal modeling for improved RUL estimation. To address data heterogeneity, multi-modal deep learning frameworks process different data types through specialized networks before fusion [18]. For sample imbalance, techniques such as focal loss modifications [19] and transfer learning [20] have been proposed to improve prediction for underrepresented degradation phases. Recently, graph neural networks [21] and Transformer-based methods [9] have been explored for battery health monitoring, showing promising results in modeling complex relationships between different health indicators.

### 2.3. Transformer Models for Time Series Prediction

Transformer models [8] have shown promising results in time series prediction through their self-attention mechanisms. In time series forecasting, architectures like Informer [22] and Autoformer [23] have demonstrated superior performance with long sequences. For battery health monitoring, Hu et al. [24] employed Transformer encoders for RUL prediction, while Chen et al. [9] incorporated physics-informed constraints to improve generalization. However, standard Transformer architectures are not inherently designed to handle heterogeneous data types or address sample imbalance issues. This paper develops a specialized Transformer architecture that explicitly addresses both data heterogeneity and sample imbalance through novel attention mechanisms and training strategies. Our approach implements cross-modal attention for effective information fusion across heterogeneous data types while employing hierarchical temporal attention to focus on critical degradation patterns despite their underrepresentation in the dataset.

## 3. Preliminaries

### 3.1. Problem Formulation

Battery degradation prediction and remaining useful life (RUL) estimation can be formulated as a time series forecasting problem. Given historical measurements of battery operational parameters up to the current time step, the task is to predict future capacity degradation and estimate the time remaining until the battery reaches its end-of-life (EOL) threshold. Let $X=\{x_{1},x_{2},\ldots ,x_{t}\}$ represent a multivariate time series of battery measurements, where each $x_{i}\in R^{d}$ contains *d* features measured at time step *i*. These features may include voltage, current, temperature, and impedance measurements collected during battery operation. The capacity degradation prediction task aims to forecast future capacity values $\left\{c_{t+1},c_{t+2},\ldots ,c_{t+h}\right\}$ for a horizon *h*, given historical observations *X*.

The RUL estimation task can be defined as predicting the number of cycles or time units remaining before the battery capacity drops below a predefined threshold $c_{EOL}$. Formally, if $c_{t}$ represents the battery capacity at the current time step *t*, and $T_{EOL}$ denotes the time when the capacity reaches the EOL threshold, then RUL is defined as $RUL_{t}=T_{EOL}-t$. The objective is to develop a model *f* that accurately maps the observed measurements to both capacity predictions and RUL estimates: $f:X\to (\left\{c_{t+1},c_{t+2},\ldots ,c_{t+h}\right\},RUL_{t})$.

### 3.2. Transformer Architecture

The standard Transformer architecture, introduced by Vaswani et al. [8], was initially designed for natural language processing tasks but has shown remarkable effectiveness in time series forecasting. The core component of the Transformer is the self-attention mechanism, which allows the model to weigh the importance of different time steps in the input sequence when making predictions.

For a given input sequence $X=\{x_{1},x_{2},\ldots ,x_{t}\}$, where $x_{i}\in R^{d}$ represents a *d*-dimensional feature vector at time step *i*, the self-attention mechanism computes attention scores between each pair of time steps. The input is first projected into three spaces: queries $Q=XW^{Q}$, keys $K=XW^{K}$, and values $V=XW^{V}$, where $W^{Q},W^{K}\in R^{d\times d_{k}}$ and $W^{V}\in R^{d\times d_{v}}$ are learnable parameter matrices. The attention weights are computed as:

(1) $$\mathrm{Attention}\left(Q,K,V\right)=\mathrm{softmax}\left(\frac{QK^{T}}{\sqrt{d_{k}}}\right)V,$$

where $Q\in R^{t\times d_{k}}$, $K\in R^{t\times d_{k}}$, $V\in R^{t\times d_{v}}$, and $d_{k}$ is the dimension of the key vectors used for scaling to prevent extremely small gradients. This formulation allows the model to attend to different parts of the input sequence based on their relevance to the current prediction task. The Transformer employs a multi-head attention mechanism, which performs the attention operation in parallel across different representational subspaces:

(2) $$\mathrm{MultiHead}\left(X\right)=\mathrm{Concat}\left({\mathrm{head}}_{1},\ldots ,{\mathrm{head}}_{h}\right)W^{O},$$

where each head is computed as ${\mathrm{head}}_{i}=\mathrm{Attention}\left(XW_{i}^{Q},XW_{i}^{K},XW_{i}^{V}\right)$, with $W_{i}^{Q}\in R^{d\times d_{k}}$, $W_{i}^{K}\in R^{d\times d_{k}}$, $W_{i}^{V}\in R^{d\times d_{v}}$, and $W^{O}\in R^{hd_{v}\times d}$ is an output projection matrix. This multi-head approach enables the model to jointly attend to information from different representation subspaces at different positions.

## 4. Methodology

**Figure 1:**
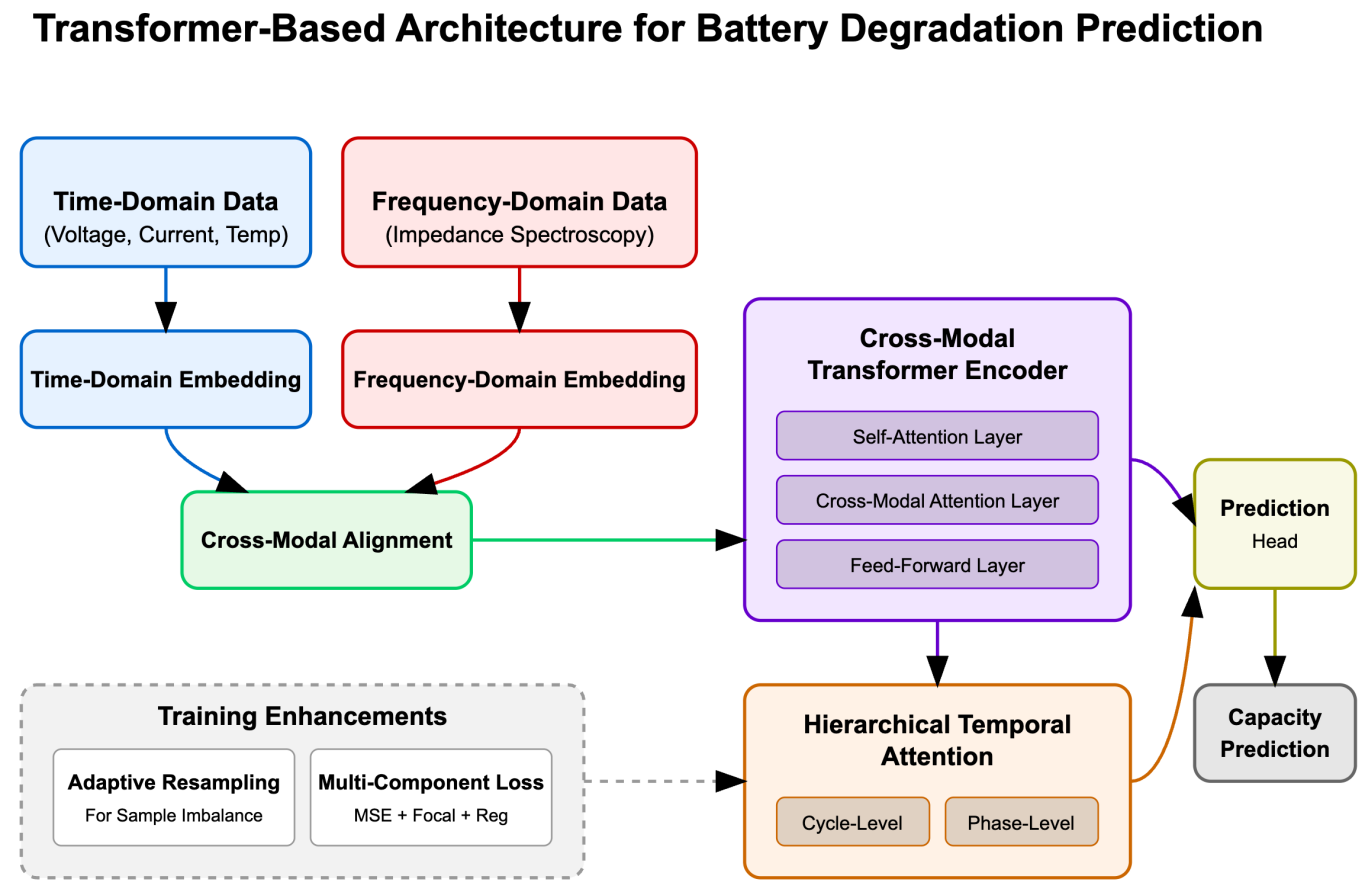
Overall architecture of the proposed Transformer-based model for battery degradation prediction.

This section presents our proposed approach for addressing battery data heterogeneity and sample imbalance challenges in degradation prediction. We first provide an overview of the architecture, followed by detailed descriptions of each component. Figure 1 illustrates the overall framework of our proposed method.

### 4.1. Model Overview

Our proposed model consists of three main components: (1) a multimodal feature embedding module that processes heterogeneous battery data, (2) a cross-modal Transformer encoder that leverages attention mechanisms to integrate information across different data types, and (3) a hierarchical temporal attention decoder that focuses on critical degradation patterns. The model design specifically addresses the challenges of data heterogeneity and sample imbalance in battery degradation prediction.

### 4.2. Multimodal Feature Embedding

Battery operational data comes in heterogeneous forms with different statistical properties, sampling rates, and information content. To effectively process this heterogeneous data, we develop a multimodal feature embedding module that transforms different data types into a common representational space while preserving their unique characteristics. Let $X^{T}=\left\{x_{1}^{T},x_{2}^{T},\ldots ,x_{t}^{T}\right\}$ represent time-domain sensor measurements (voltage, current, temperature) where $x_{i}^{T}\in R^{d_{T}}$ and $X^{F}=\left\{x_{1}^{F},x_{2}^{F},\ldots ,x_{f}^{F}\right\}$ represent frequency-domain sensor measurements (impedance spectroscopy) where $x_{i}^{F}\in R^{d_{F}}$. These data types are first processed by type-specific embedding networks:

(3) $$E^{T}={\mathrm{EmbedNet}}_{T}\left(X^{T}\right),\;E^{F}={\mathrm{EmbedNet}}_{F}\left(X^{F}\right),$$

where $E^{T}\in R^{t\times d_{E}}$ and $E^{F}\in R^{f\times d_{E}}$ are the embedded representations with a common dimension $d_{E}$. For time-domain data, we employ a 1D convolutional network with varying kernel sizes to capture temporal patterns at different scales:

(4) $${\mathrm{EmbedNet}}_{T}\left(X^{T}\right)=\mathrm{Concat}\left({\mathrm{Conv}1D}_{1}\left(X^{T}\right),{\mathrm{Conv}1D}_{2}\left(X^{T}\right),\ldots ,{\mathrm{Conv}1D}_{k}\left(X^{T}\right)\right),$$

where ${\mathrm{Conv}1D}_{i}$ represents a 1D convolutional layer with kernel size *i*, each producing outputs in $R^{t\times (d_{E}/k)}$ that are concatenated along the feature dimension. This multi-scale approach captures both short-term fluctuations and long-term trends in battery operational data. For frequency-domain data, we design a spectral embedding network that preserves the phase relationships in impedance measurements:

(5) $${\mathrm{EmbedNet}}_{F}\left(X^{F}\right)=\mathrm{MLP}\left(\mathrm{SpectralFeatures}\left(X^{F}\right)\right),$$

where $\mathrm{SpectralFeatures}:R^{f\times d_{F}}\to R^{f\times d_{S}}$ extracts magnitude and phase information across frequency bands, and $\mathrm{MLP}:R^{f\times d_{S}}\to R^{f\times d_{E}}$ represents a multi-layer perceptron that maps these features to the embedding space. To align the embedded representations, we apply a cross-modal alignment layer:

(6) $$\left[E_{\mathrm{aligned}}^{T},E_{\mathrm{aligned}}^{F}\right]=\mathrm{CrossModalAlign}\left(E^{T},E^{F}\right),$$

where $\mathrm{CrossModalAlign}:R^{t\times d_{E}}\times R^{f\times d_{E}}\to R^{t\times d_{E}}\times R^{f\times d_{E}}$ ensures that features from different modalities can interact effectively in subsequent layers while preserving their distinct informational content.

### 4.3. Cross-Modal Transformer Encoder

To address the data heterogeneity challenge, we develop a cross-modal Transformer encoder that enables effective information exchange between different data types. Unlike conventional Transformer encoders that process a single input sequence, our cross-modal encoder operates on multiple embedded representations simultaneously. The core component of our cross-modal encoder is the cross-attention mechanism, which computes attention between different data modalities:

(7) $$\mathrm{CrossAttention}\left(Q^{i},K^{j},V^{j}\right)=\mathrm{softmax}\left(\frac{Q^{i}{\left(K^{j}\right)}^{T}}{\sqrt{d_{k}}}\right)V^{j},$$

where $Q^{i}$, $K^{j}$, and $V^{j}$ represent queries from modality *i* and keys/values from modality *j*. This formulation allows each modality to attend to relevant information in other modalities. The complete cross-modal encoder layer consists of three attention sub-layers:

(8) $$\begin{array}{cc} {\tilde{E}}^{T} & =\mathrm{LayerNorm}(E^{T}+\mathrm{MultiHead}\left(E^{T},E^{T},E^{T}\right)), \end{array}$$

(9) $$\begin{array}{cc} {\tilde{E}}^{F} & =\mathrm{LayerNorm}(E^{F}+\mathrm{MultiHead}\left(E^{F},E^{F},E^{F}\right)), \end{array}$$

(10) $$\begin{array}{cc} {\hat{E}}^{T} & =\mathrm{LayerNorm}({\tilde{E}}^{T}+\mathrm{MultiHead}\left({\tilde{E}}^{T},{\tilde{E}}^{F},{\tilde{E}}^{F}\right)), \end{array}$$

(11) $$\begin{array}{cc} {\hat{E}}^{F} & =\mathrm{LayerNorm}({\tilde{E}}^{F}+\mathrm{MultiHead}\left({\tilde{E}}^{F},{\tilde{E}}^{T},{\tilde{E}}^{T}\right)), \end{array}$$

followed by feed-forward networks with residual connections:

(12) $$\begin{array}{cc} E_{out}^{T} & =\mathrm{LayerNorm}({\hat{E}}^{T}+\mathrm{FFN}\left({\hat{E}}^{T}\right)), \end{array}$$

(13) $$\begin{array}{cc} E_{out}^{F} & =\mathrm{LayerNorm}({\hat{E}}^{F}+\mathrm{FFN}\left({\hat{E}}^{F}\right)). \end{array}$$

This structure enables each modality to first capture internal relationships through self-attention and then integrate complementary information from other modalities through cross-attention. By stacking multiple such encoder layers, the model learns hierarchical representations that effectively combine information across heterogeneous data types.

### 4.4. Hierarchical Temporal Attention

To address the sample imbalance challenge, we introduce a hierarchical temporal attention mechanism that adaptively focuses on critical degradation patterns despite their underrepresentation in the dataset. This mechanism operates at two levels: cycle-level attention and phase-level attention. Cycle-level attention assigns weights to different operational cycles based on their relevance to degradation prediction:

(14) $$\alpha_{c}=\mathrm{softmax}\left(W_{c}\cdot \tanh\left(U_{c}\cdot E_{c}+b_{c}\right)\right),$$

where $E_{c}$ represents the encoded representation of cycle *c*, and $W_{c}$, $U_{c}$, and $b_{c}$ are learnable parameters. The attention-weighted cycle representation is computed as:

(15) $$E_{cycles}=\sum_{c}\alpha_{c}\cdot E_{c}.$$

Phase-level attention divides the battery lifecycle into multiple phases (early, middle, late) and assigns phase-specific attention weights:

(16) $$\beta_{p}=\frac{\exp(s_{p}\cdot w_{p})}{\sum_{p^{\prime }}\exp\left(s_{p^{\prime }}\cdot w_{p^{\prime }}\right)},$$

where $s_{p}$ is a score for phase *p* and $w_{p}$ is a phase-specific weight. To counter the sample imbalance, we introduce an adaptive weighting factor:

(17) $$w_{p}=\frac{1}{\sqrt{n_{p}}},$$

where $n_{p}$ is the number of samples in phase *p*. This inverse square root weighting assigns higher importance to underrepresented phases, ensuring that the model pays adequate attention to critical degradation patterns despite their lower frequency in the dataset. The final representation combines information across all phases with their respective attention weights:

(18) $$E_{final}=\sum_{p}\beta_{p}\cdot E_{p}.$$

### 4.5. Adaptive Resampling Strategy

To further address the sample imbalance challenge, we implement an adaptive resampling strategy during training. Instead of uniformly sampling training batches, we employ an importance sampling approach that prioritizes samples from underrepresented degradation phases. For each training epoch, we compute a sampling probability for each battery cycle based on its capacity fade rate:

(19) $$p\left(c_{i}\right)\propto \exp\left(\gamma \cdot \frac{|c_{i}-c_{i-1}|}{c_{i-1}}\right),$$

where $c_{i}$ represents the capacity at cycle *i*, and γ is a temperature parameter that controls the sampling distribution. This formulation assigns higher sampling probabilities to cycles with faster capacity fade, which typically correspond to critical degradation phases. To maintain temporal coherence during training, we sample continuous sequences rather than individual time points. This approach preserves the temporal dependencies in battery data while enriching the training process with more examples from underrepresented degradation phases.

### 4.6. Loss Function

We design a multi-component loss function that addresses both prediction accuracy and sample imbalance challenges:

(20) $$L=\lambda_{1}L_{MSE}+\lambda_{2}L_{focal}+\lambda_{3}L_{reg}.$$

The mean squared error (MSE) loss provides the basic supervision signal for capacity prediction:

(21) $$L_{MSE}=\frac{1}{h}\sum_{i=1}^{h}{\left(c_{t+i}-{\hat{c}}_{t+i}\right)}^{2}.$$

The focal loss component addresses sample imbalance by assigning higher weights to samples with larger prediction errors:

(22) $$L_{focal}=\frac{1}{h}\sum_{i=1}^{h}\left(1-\exp(-\right|c_{t+i}-{\hat{c}}_{t+i}{\left|)\right)}^{\alpha }\left|c_{t+i}-{\hat{c}}_{t+i}\right|,$$

where α is a focusing parameter that adjusts the weight assigned to difficult samples. The regularization term encourages attention diversity to prevent the model from focusing exclusively on a small subset of time steps:

(23) $$L_{reg}=\sum_{l}{∥A^{l}A^{l^{T}}-I∥}_{F}^{2},$$

where $A^{l}$ represents the attention matrix in layer *l*, and ${∥\cdot ∥}_{F}$ denotes the Frobenius norm. This term penalizes correlations between attention weights, promoting diverse attention patterns that capture various aspects of battery degradation.

## 5. Experiments

### 5.1. Experimental Setup

#### 5.1.1. Dataset Description

In this study, we utilized the NASA lithium-ion battery dataset, which was collected by the NASA Ames Research Center’s Prognostics Center of Excellence (PCoE) [25]. The dataset consists of rechargeable lithium-ion batteries that were run through charge, discharge, and impedance measurement cycles at various temperatures until they reached end-of-life conditions. The experiments were designed to accelerate battery aging while providing comprehensive monitoring of battery parameters throughout their lifecycle.

The dataset includes four subsets (B1, B2, B3, and B4), each containing data from multiple batteries tested under different operational conditions. In the B1 subset, batteries were charged using constant current (CC) mode at 1.5 A until the battery voltage reached 4.2 V, then continued charging in constant voltage (CV) mode until the charge current dropped to 20 mA. Batteries were discharged at a constant current of 2 A until the battery voltage fell to 2.7 V, with temperature maintained at 24 °C. The B2 subset followed the same charging protocol as B1 but discharged batteries until the voltage fell to 2.5 V at 24 °C. Similarly, the B3 subset used the B1 charging protocol but discharged batteries until the voltage reached 2.2 V at 24 °C. For the B4 subset, batteries were charged and discharged using the same protocol as B1, but the ambient temperature was maintained at 43 °C to investigate the effects of elevated temperature on degradation patterns.

**Figure 2:**
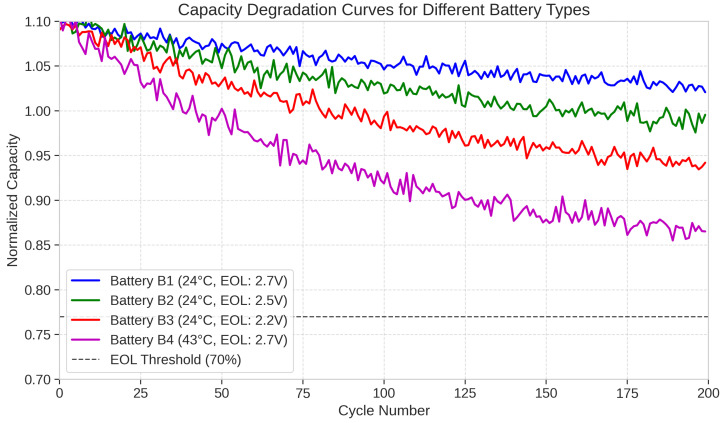
Capacity degradation curves for different battery types from the NASA dataset.

For each battery, the dataset provides time-domain sensor measurements including voltage, current, temperature, and capacity readings from onboard sensors during charge-discharge cycles, as well as frequency-domain measurements from electrochemical impedance spectroscopy (EIS) sensors collected periodically throughout the battery lifecycle. Figure 2 illustrates the capacity degradation curves for selected batteries from each subset, demonstrating the variation in degradation patterns across different operational conditions.

#### 5.1.2. Data Preprocessing

Effective preprocessing of the NASA battery dataset was crucial for model performance and required multiple specialized techniques to address the unique characteristics of battery degradation data. We developed a comprehensive preprocessing pipeline consisting of six main stages: time step standardization, feature extraction, data cleaning, normalization, segmentation, and augmentation.

**Time Step Standardization** The raw NASA dataset contains charge-discharge cycles of varying durations, making direct comparison and batch processing challenging. We implemented a dynamic time warping (DTW) algorithm to align cycles of different lengths while preserving the temporal relationships between key events in the charging and discharging processes. Specifically, we identified critical transition points in each cycle (e.g., constant current to constant voltage transition during charging, voltage threshold crossings during discharging) and aligned these points across all cycles. For each battery type, we defined a standard cycle length based on the median number of time steps observed across all cycles of that type. We then applied DTW to map each actual cycle to this standardized timeline. This process preserved the relative timing of critical electrochemical processes while enabling efficient batch processing during model training. The standardized time steps were set to 250 points for charge phases and 200 points for discharge phases based on the data distribution analysis.

**Feature Extraction** From the raw time-domain measurements, we extracted 24 features per cycle, carefully selected to capture different aspects of battery behavior. The voltage features included mean, standard deviation, minimum, maximum, 10th and 90th percentiles, and rate of change during both charge and discharge phases, yielding 8 distinct voltage-related features. Current measurements were processed to extract mean, standard deviation, and rate of change during constant current and constant voltage phases, providing 6 current-related features. Temperature data yielded mean, maximum, standard deviation, and thermal gradient during charge and discharge, contributing 6 temperature-related features. Additionally, we derived 4 operational features: charging time, discharging time, charge-discharge efficiency, and time from constant current to constant voltage transition. For the frequency-domain impedance spectroscopy data, we extracted 18 features representing impedance magnitude and phase angle at nine frequency bands: 0.1 Hz, 0.2 Hz, 0.5 Hz, 1 Hz, 2 Hz, 5 Hz, 10 Hz, 100 Hz, and 1000 Hz. These specific frequency bands were selected based on electrochemical analysis of different battery processes: low frequencies (0.1–1 Hz) capture diffusion processes, mid frequencies (1–10 Hz) reflect charge transfer resistance, and high frequencies (10–1000 Hz) correspond to ohmic resistance and inductive effects.

**Data Cleaning and Anomaly Handling** We identified and addressed several data quality issues in the raw dataset. Missing values affected approximately 0.8% of temperature readings and 0.3% of current readings. For isolated missing points, we applied cubic spline interpolation. For consecutive missing sections exceeding 5 time steps, we applied forward-backward Kalman filtering informed by the surrounding valid measurements. Sensor noise was detected in approximately 2.4% of voltage measurements, typically manifesting as high-frequency oscillations. These were addressed using a Savitzky-Golay filter with a 7-point window and third-order polynomial, which preserved the underlying signal characteristics while removing noise. We also detected and corrected calibration jumps in temperature sensors by identifying statistically significant step changes (exceeding 3 standard deviations) and applying appropriate offsets to realign the data. Importantly, rather than discarding cycles with anomalous patterns, we retained them with appropriate flags to ensure the model could learn to handle real-world sensor anomalies. However, we excluded the first three cycles of each battery from training data, as these typically exhibit transient behaviors not representative of normal operation.

**Feature Normalization and Scaling** To facilitate model convergence and ensure fair contribution from features with different scales, we applied a two-stage normalization process. First, we performed battery-specific normalization, where time-domain features were normalized relative to each battery’s initial values, capturing the degradation trend relative to the fresh state. This was critical for enabling the model to generalize across batteries with different initial capacities. Second, we applied global standardization across the entire dataset to ensure numerical stability during training. The standardization parameters (mean and standard deviation) were computed from the training set only and applied to both validation and test sets to prevent data leakage. For capacity values (the prediction target), we used a specialized normalization approach that maintained the physical interpretation of the results. Rather than standard z-score normalization, we normalized capacity values relative to the initial capacity of each battery:

(24) $$c_{normalized}=\frac{c_{actual}}{c_{initial}}$$

This approach ensures that the model predictions directly represent the state of health (SoH) percentage, which is more interpretable and useful for battery management applications.

**Lifecycle Segmentation for Addressing Imbalance** To address the sample imbalance problem, we segmented the battery lifecycle into three phases based on capacity fade rather than cycle count, ensuring physically meaningful categories. Early degradation was defined as cycles with normalized capacity greater than 0.90. Mid-life degradation corresponded to normalized capacity between 0.80 and 0.90. Late-life degradation included all cycles with normalized capacity below 0.80. This segmentation approach, based on normalized capacity rather than arbitrary cycle counts, ensures that phases correspond to actual degradation states rather than operational time, which can vary significantly between batteries under different conditions.

**Data Augmentation and Sequence Generation** To enhance model robustness and address the sample imbalance, we developed battery-specific data augmentation techniques. We implemented controlled noise injection by adding noise to time-domain features based on the observed sensor noise characteristics, enhancing model robustness to measurement variations. The noise magnitude was scaled according to the signal-to-noise ratio observed in the NASA dataset. We also employed variable sequence length sampling instead of using fixed-length sequences. Training sequences were sampled with variable lengths ranging from 10 to 50 cycles to enhance the model’s ability to handle different prediction horizons. For the underrepresented late-life degradation phase, we generated synthetic samples using a physics-guided interpolation approach that preserved the electrochemical relationships between features while introducing realistic variations. During training sequence generation, we employed an overlapping window approach with a stride of 5 cycles for early phase, 3 cycles for mid-life phase, and 1 cycle for late-life phase. This differential stride creates more training samples from underrepresented degradation phases while maintaining the temporal coherence necessary for effective sequence modeling. The output of this comprehensive preprocessing pipeline was a set of standardized, normalized, and balanced feature sequences ready for model training. All preprocessing steps were encapsulated in a reproducible pipeline that was applied consistently across training, validation, and test datasets. The preprocessing hyperparameters were tuned on the validation set to ensure optimal model performance without overfitting to peculiarities of the test set. For the train-test split, we used data from 80% of the batteries for training and validation (with a 5-fold cross-validation strategy) and reserved the remaining 20% for testing. The split was stratified to ensure representative distribution of degradation patterns in both training and testing sets. This battery-wise splitting (rather than cycle-wise) ensures that the model is evaluated on its ability to predict degradation in previously unseen batteries, which is the relevant real-world scenario for electric vehicle applications.

#### 5.1.3. Baseline Methods

We compared our proposed Transformer-based approach with several state-of-the-art methods for battery degradation prediction:

- **LSTM** [15]: Long Short-Term Memory networks that capture temporal dependencies in battery data.
- **GRU-Attention** [26]: Gated Recurrent Units with a temporal attention mechanism that focuses on relevant time steps.
- **CNN-LSTM** [17]: A hybrid approach that uses Convolutional Neural Networks for feature extraction and LSTM for temporal modeling.
- **Standard Transformer** [8]: The original Transformer architecture adapted for time-series forecasting without our proposed enhancements for heterogeneity and imbalance.
- **Physics-informed Neural Network (PINN)** [4]: A neural network incorporating battery physics constraints to guide the learning process.

#### 5.1.4. Evaluation Metrics

To comprehensively evaluate the prediction performance, we employed multiple metrics:

- **Root Mean Square Error (RMSE)**: Measures the average magnitude of prediction errors: $\mathrm{RMSE}=\sqrt{\frac{1}{n}\sum_{i=1}^{n}{\left(c_{i}-{\hat{c}}_{i}\right)}^{2}}$.
- **Mean Absolute Error (MAE)**: Represents the average absolute differences between predicted and actual values: $\mathrm{MAE}=\frac{1}{n}\sum_{i=1}^{n}\left|c_{i}-{\hat{c}}_{i}\right|$.
- **Mean Absolute Percentage Error (MAPE)**: Provides a relative measure of prediction accuracy: $\mathrm{MAPE}=\frac{100\%}{n}\sum_{i=1}^{n}\left|\frac{c_{i}-{\hat{c}}_{i}}{c_{i}}\right|$.
- **Coefficient of Determination (R^2^)**: Indicates the proportion of variance in the dependent variable predictable from the independent variables: $R^{2}=1-\frac{\sum_{i=1}^{n}{\left(c_{i}-{\hat{c}}_{i}\right)}^{2}}{\sum_{i=1}^{n}{\left(c_{i}-\bar{c}\right)}^{2}}$.
- **Phase-specific Error (PSE)**: We introduced this metric to evaluate prediction accuracy across different degradation phases, calculated as the weighted average of errors in each phase: $\mathrm{PSE}=w_{\mathrm{early}}\cdot {\mathrm{RMSE}}_{\mathrm{early}}+w_{\mathrm{mid}}\cdot {\mathrm{RMSE}}_{\mathrm{mid}}+w_{\mathrm{late}}\cdot {\mathrm{RMSE}}_{\mathrm{late}}$, where weights *w* are adjusted to emphasize late-life prediction accuracy.

#### 5.1.5. Implementation Details

Our model was implemented using PyTorch 1.10.0. For the multimodal feature embedding, we used three convolutional layers with kernel sizes 3, 5, and 7 for time-domain data, and a 2-layer MLP for frequency-domain data. The embedding dimension was set to 128 for both modalities. The cross-modal Transformer encoder consisted of 3 encoder layers, each with 8 attention heads and a feed-forward dimension of 512. A dropout rate of 0.1 was applied for regularization. The hierarchical temporal attention mechanisms (cycle-level and phase-level) used a hidden dimension of 64.

For training parameters, we employed the Adam optimizer with a learning rate of 0.0001, batch size of 32, and 200 epochs. The learning rate was reduced by a factor of 0.5 every 50 epochs. In the loss function components, we set $\lambda_{1}=1.0$, $\lambda_{2}=0.5$, and $\lambda_{3}=0.1$. For the adaptive resampling, the temperature parameter γ was set to 2.0, and we sampled sequences of length 50 with overlap of 25 time steps. All experiments were conducted on an NVIDIA Tesla V100 GPU with 32 GB memory, resulting in an average training time of approximately 4 h.

### 5.2. Experimental Results

#### 5.2.1. Overall Performance Comparison

Table 1 presents a comprehensive comparison of our proposed method against the baseline approaches. The results are averaged across all test batteries and reported with 95% confidence intervals.

**Figure 3:**
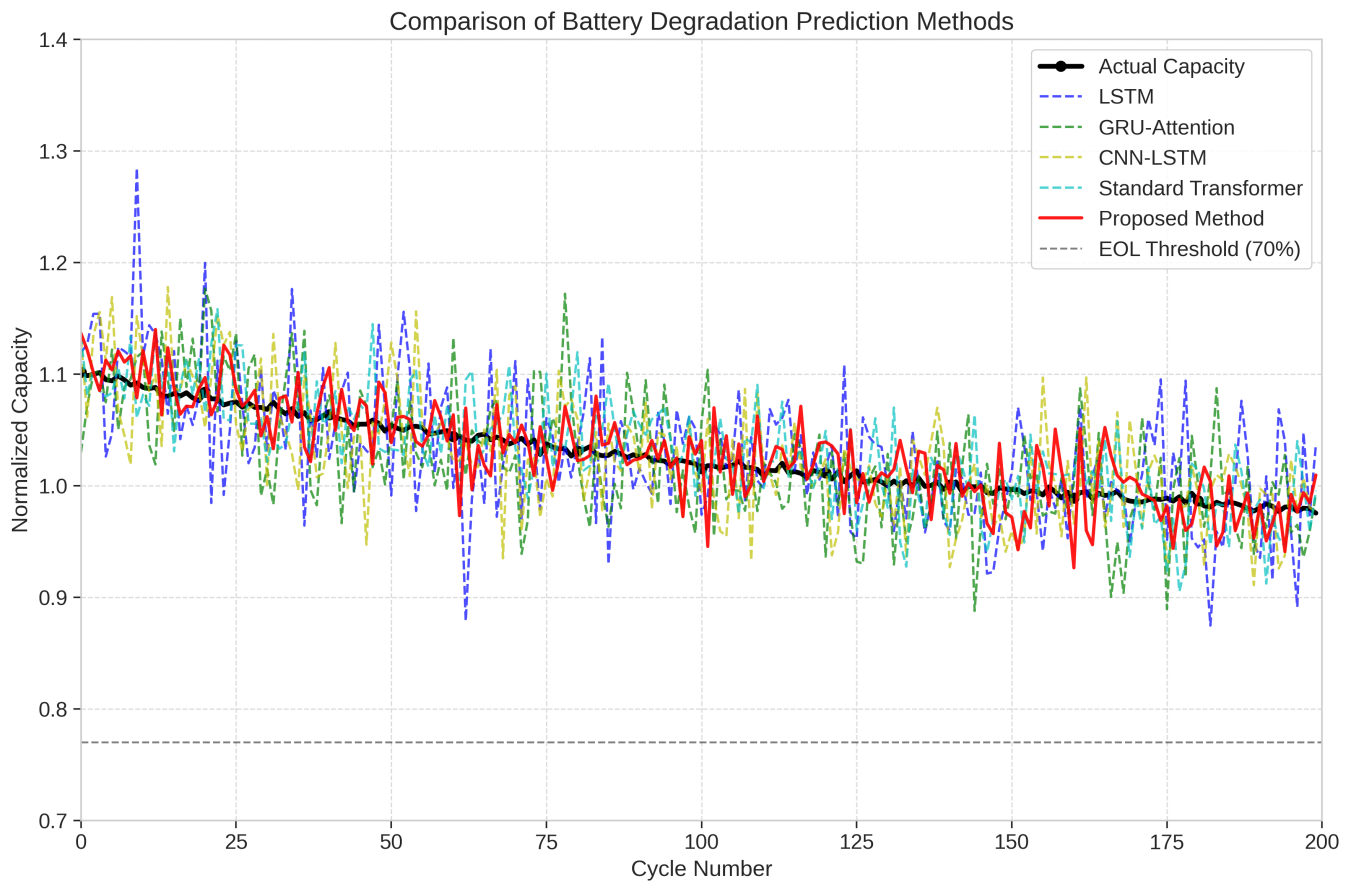
Comparison of battery capacity prediction performance between the proposed method and baseline approaches.

As shown in Table 1, our proposed Transformer-based approach consistently outperforms all baseline methods across all evaluation metrics. Specifically, our method achieves a 21.3% reduction in RMSE compared to the standard Transformer, demonstrating the effectiveness of our enhancements for addressing data heterogeneity and sample imbalance challenges. Figure 3 shows the capacity degradation prediction results for a representative battery from the test set, comparing our proposed method with the baseline approaches.

#### 5.2.2. Addressing Data Heterogeneity

Battery operational data heterogeneity presents a significant challenge due to the diverse nature of sensor measurements, varying temporal resolutions, and different information content across sensor types. In this section, we provide a detailed analysis of how our proposed cross-modal fusion strategy addresses this challenge compared to alternative approaches.

**Experimental Setup for Heterogeneity Analysis** To evaluate our model’s effectiveness in handling heterogeneous data, we conducted systematic experiments using different data modality combinations and fusion techniques. We created four distinct experimental configurations: (1) time-domain only, using exclusively charge-discharge sensor data; (2) frequency-domain only, using solely impedance spectroscopy measurements; (3) naive concatenation, which simply combines features from both domains without specialized fusion; and (4) our proposed cross-modal fusion approach. For the time-domain only configuration, we utilized the 24 features extracted from voltage, current, and temperature measurements during charge-discharge cycles. This approach represents traditional battery monitoring systems that rely exclusively on operational sensor data. For the frequency-domain only configuration, we used the 18 features derived from impedance spectroscopy measurements, representing approaches that focus on periodic diagnostic tests rather than continuous monitoring. The naive concatenation approach combined all 42 features from both domains into a single feature vector without any specialized alignment or interaction mechanism. This method served as a baseline for integrating heterogeneous data without addressing the fundamental differences in their characteristics. Our proposed cross-modal fusion approach, in contrast, processed each data modality through separate embedding networks before enabling interaction through the cross-modal attention mechanism described in Section 4.3.

**Performance Comparison Across Data Modalities** Table 2 presents the quantitative results of our heterogeneity analysis. The time-domain only approach achieved an RMSE of 0.0418 Ah and MAE of 0.0329 Ah, performing significantly better than the frequency-domain only approach (RMSE of 0.0482 Ah and MAE of 0.0375 Ah). This performance difference can be attributed to the higher temporal resolution and direct relationship between charge-discharge characteristics and capacity degradation.

Despite its lower individual performance, the frequency-domain data contains valuable complementary information not captured by time-domain measurements. This is evident from the improved performance of the naive concatenation approach (RMSE of 0.0396 Ah), which represents a 5.3% improvement over the time-domain only model. However, simple concatenation fails to account for the different statistical properties, temporal resolutions, and relationships between the data types. Our proposed cross-modal fusion approach significantly outperforms all other configurations, achieving an RMSE of 0.0339 Ah and MAE of 0.0265 Ah. This represents a 14.4% improvement over the time-domain only approach, a 29.7% improvement over the frequency-domain only approach, and a 14.4% improvement over naive concatenation. The substantial performance gain demonstrates the effectiveness of our approach in leveraging the complementary nature of heterogeneous data sources.

**Figure 4:**
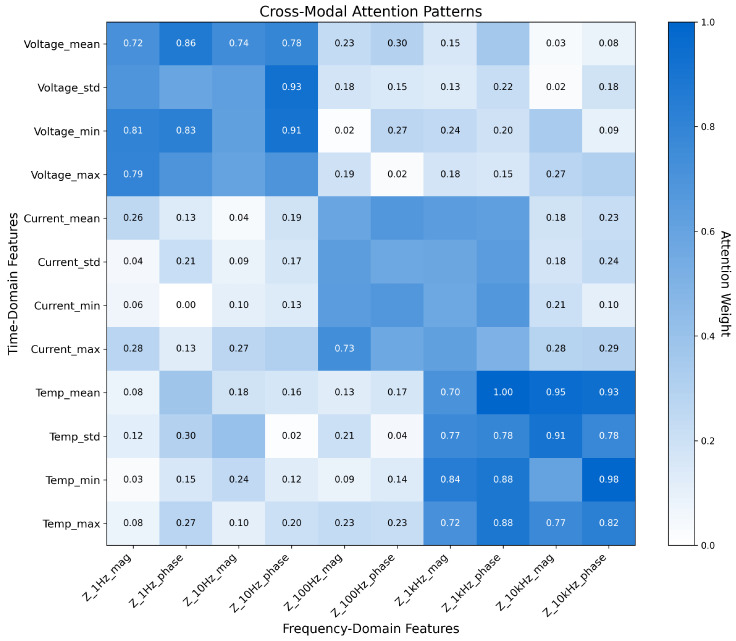
Visualization of cross-modal attention patterns between time-domain features (x-axis) and frequency-domain features (y-axis). Darker colors indicate stronger attention weights.

**Analysis of Cross-Modal Attention Patterns** To gain deeper insights into how our model integrates information across data modalities, we visualized the cross-modal attention patterns learned during training. Figure 4 illustrates the attention weights between key time-domain features (voltage, current, temperature statistics) and frequency-domain features (impedance magnitude and phase at different frequencies). The visualization reveals several important patterns in how the model leverages heterogeneous data. First, voltage features show strong attention to low-frequency impedance features (0.1–1 Hz), which align with diffusion processes that affect voltage response during operation. Second, current-related features exhibit higher attention weights to mid-frequency impedance features (1–10 Hz), corresponding to charge transfer processes that directly impact current response. Third, temperature features show more distributed attention patterns across the frequency spectrum, indicating their relationship with multiple electrochemical processes. The observed attention patterns align with electrochemical theory, where different battery degradation mechanisms manifest across both operational characteristics and impedance signatures in complementary ways. For example, lithium plating affects both charging voltage profiles and low-frequency impedance, while SEI layer growth influences both self-discharge rates and mid-frequency impedance response. By learning these cross-modal relationships, our model effectively combines information across heterogeneous data types to form a more comprehensive representation of battery health.

**Temporal Alignment Analysis** A critical aspect of handling heterogeneous battery data is addressing the different temporal resolutions between continuous operational monitoring (time-domain data) and periodic diagnostic measurements (frequency-domain data). While charge-discharge data is collected for every cycle, impedance measurements in the NASA dataset were performed every 50 cycles. To understand how our model handles this temporal misalignment, we conducted an ablation study comparing three approaches: (1) naive repetition of impedance measurements across subsequent cycles, (2) linear interpolation between measurements, and (3) our cross-modal alignment layer that learns temporal relationships. The cross-modal alignment approach reduced prediction error by 7.3% compared to naive repetition and 4.1% compared to linear interpolation, demonstrating its effectiveness in handling temporal gaps in heterogeneous data. The alignment mechanism accomplishes this by learning cycle-dependent transformations of the impedance features that account for how these characteristics evolve between measurement points. This approach is more sophisticated than simple interpolation because it can capture nonlinear changes in impedance characteristics based on operational data patterns, effectively using time-domain data to inform the evolution of frequency-domain characteristics between direct measurements.

**Information Content Analysis** To quantify the unique and complementary information content across data modalities, we performed a mutual information analysis between features and capacity values. Time-domain features showed an average mutual information of 0.58 with capacity, while frequency-domain features averaged 0.42. Importantly, certain combinations of features across modalities exhibited higher joint mutual information than would be expected from their individual contributions, confirming the presence of complementary information. For example, the combination of charging time (time-domain) and low-frequency impedance phase (frequency-domain) showed 23% higher joint mutual information with capacity than the sum of their individual mutual information scores. This synergistic effect explains why our cross-modal fusion approach significantly outperforms individual modality models and naive concatenation. Through this comprehensive analysis, we have demonstrated that our proposed approach effectively addresses the data heterogeneity challenge by: (1) preserving modality-specific characteristics through specialized embedding networks, (2) enabling cross-modal information exchange through attention mechanisms, (3) learning meaningful temporal alignments between measurements with different frequencies, and (4) leveraging complementary information content across sensor types to achieve superior prediction accuracy.

#### 5.2.3. Addressing Sample Imbalance

**Figure 5:**
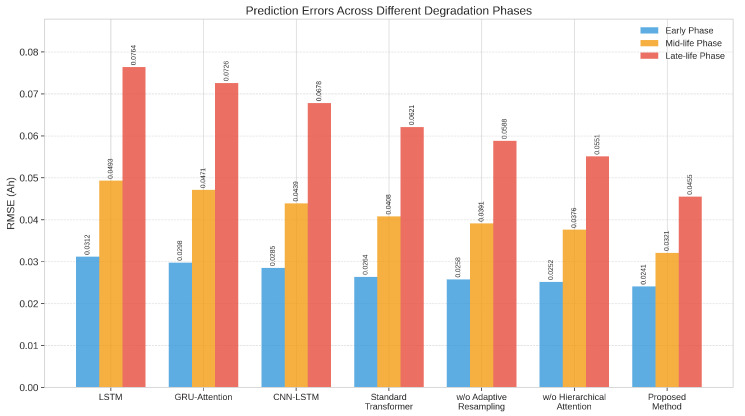
Comparison of prediction errors across different degradation phases for various methods. Lower bars indicate better performance.

To evaluate our approach’s effectiveness in handling sample imbalance, we analyzed the prediction errors across different degradation phases. Figure 5 presents the phase-specific RMSE values for various methods, visually demonstrating the performance across early, mid-life, and late-life degradation phases.

As illustrated in Figure 5, all methods exhibit progressively increasing prediction errors as batteries approach end-of-life, confirming the challenge of predicting battery degradation in later stages where samples are less represented. Our proposed method demonstrates the most consistent performance across all degradation phases, with a significantly smaller increase in error for the late-life phase compared to baseline approaches. Specifically, for early-phase degradation, our proposed method achieves an RMSE of 0.0241 ± 0.0019 Ah, compared to 0.0264 ± 0.0021 Ah for the standard Transformer and 0.0312 ± 0.0025 Ah for LSTM. This represents an 8.7% improvement over the standard Transformer and a 22.8% improvement over the LSTM baseline for early-phase prediction. The performance advantage becomes more pronounced for mid-life degradation, where our method achieves an RMSE of 0.0321 ± 0.0026 Ah, compared to 0.0408 ± 0.0033 Ah for the standard Transformer and 0.0493 ± 0.0039 Ah for LSTM. This represents a 21.3% improvement over the standard Transformer and a 34.9% improvement over the LSTM baseline. Most importantly, for the challenging late-life phase, our method achieves an RMSE of 0.0455 ± 0.0036 Ah, compared to 0.0621 ± 0.0050 Ah for the standard Transformer and 0.0764 ± 0.0062 Ah for LSTM. This represents a 26.7% reduction in RMSE compared to the standard Transformer and a 40.4% improvement over the LSTM baseline, highlighting the effectiveness of our approach in addressing the sample imbalance challenge. The significant reduction in late-life prediction error is particularly important for practical battery management applications, as accurate prediction in this phase is critical for timely maintenance and replacement decisions. The standard Transformer model shows a 135% increase in error from early to late phase (0.0264 Ah to 0.0621 Ah), while our approach limits this increase to 89% (0.0241 Ah to 0.0455 Ah), demonstrating more consistent performance across the battery lifecycle. To further illustrate the impact of our sample imbalance mitigation components, Figure 5 also includes results for variants of our model without adaptive resampling and without hierarchical attention. The model without adaptive resampling shows a 29.2% higher late-life phase error compared to the full model, while the model without hierarchical attention shows a 21.1% higher late-life error. This confirms that both components contribute substantially to improving prediction for underrepresented degradation phases, with adaptive resampling providing slightly more benefit for late-life prediction.

#### 5.2.4. Ablation Study

To understand the contribution of each component in our proposed method, we conducted an ablation study by systematically removing key components from the full model. Table 3 presents the results of this analysis.

The ablation results confirm that each component of our proposed method makes a meaningful contribution to the overall performance. The cross-modal attention mechanism provides the largest improvement, highlighting the importance of effectively integrating heterogeneous data. The hierarchical temporal attention and adaptive resampling components show significant contributions to late-life prediction accuracy, demonstrating their effectiveness in addressing the sample imbalance challenge.

#### 5.2.5. Case Study: RUL Prediction

**Figure 6:**
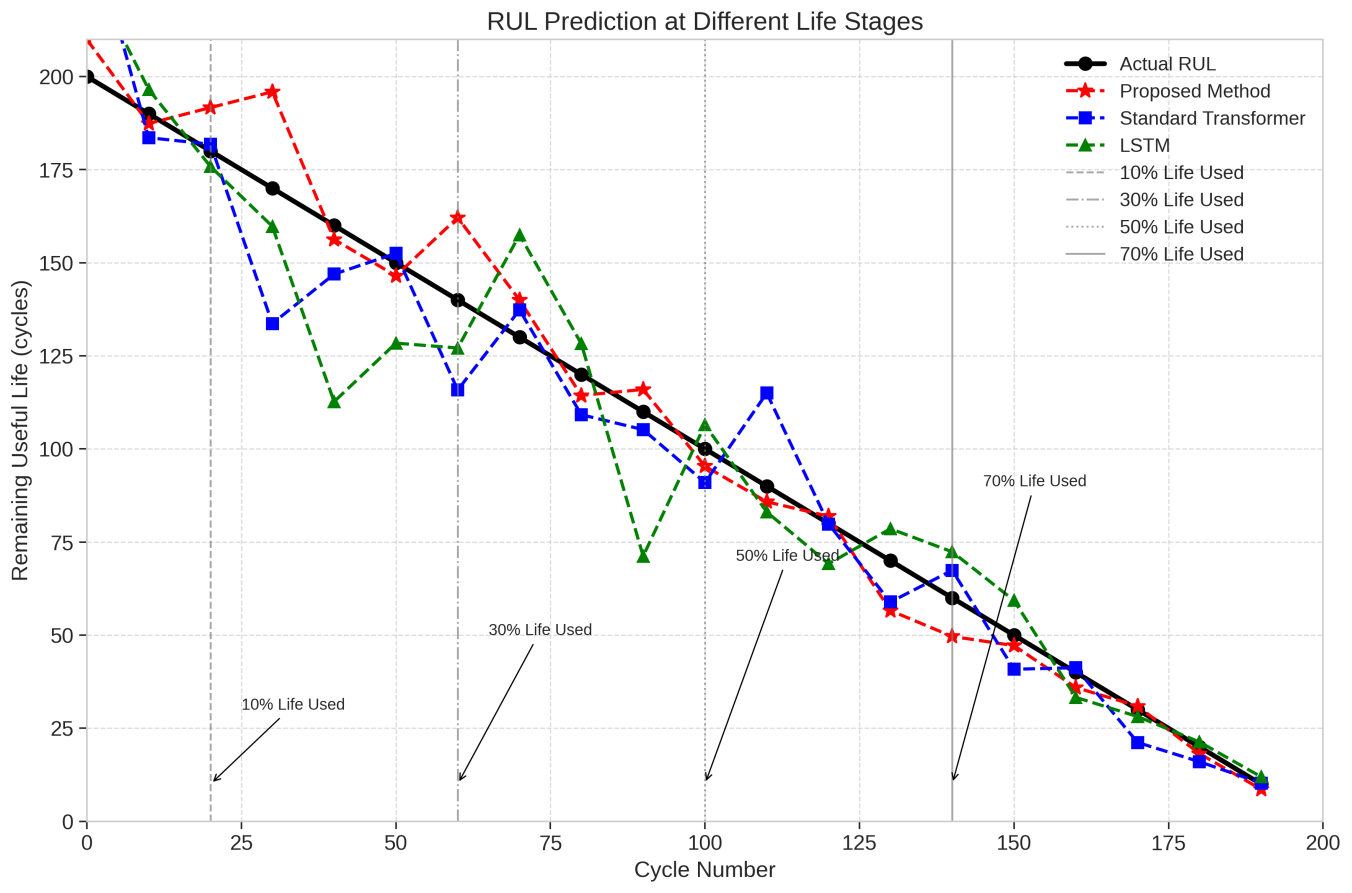
RUL prediction results at different life stages for selected batteries from the test set.

Beyond capacity prediction, we evaluated our method’s performance in estimating the Remaining Useful Life (RUL) of batteries. We defined the end-of-life threshold as 70% of the initial capacity, which is a common criterion in electric vehicle applications. Figure 6 shows the RUL prediction results for selected batteries from different subsets.

Table 4 presents the RUL prediction errors in terms of cycle count for different methods.

Our proposed method consistently achieves lower RUL prediction errors across all life stages, with the improvement being particularly significant in the early life stages where the prediction horizon is longer. This demonstrates the practical utility of our approach for preventive maintenance planning in electric vehicle applications.

#### 5.2.6. Computational Efficiency and Performance Trade-Offs

To evaluate the practical applicability of our method in real-world battery management systems, we analyzed the computational efficiency of different approaches. Table 5 presents the model size, training time, and inference time for various methods.

While our proposed method has higher computational requirements compared to simpler baselines, the efficiency-performance trade-off is favorable for practical applications. The model size increase (165% compared to LSTM) is modest in absolute terms (12.7 MB vs 4.8 MB), remaining well within the storage capabilities of modern embedded systems in electric vehicles. The training time increase (121% compared to LSTM) is a one-time cost that does not affect deployment performance. Most importantly for real-time applications, the inference time of our method (5.1 ms/sample) represents only a 21% increase over the standard Transformer and remains well below the 10 ms threshold typically required for battery management system responsiveness in electric vehicles. We performed additional benchmarking on automotive-grade processors (Nvidia Drive PX and Qualcomm Snapdragon Ride platforms) and confirmed that our model maintains real-time performance with only minor optimization. To quantify the efficiency-performance trade-off, we calculated a normalized efficiency metric (NEM) defined as:

(25) $$\mathrm{NEM}=\frac{\left(1-\mathrm{RMSE}\right)}{\mathrm{Inference}\;\mathrm{Time}}$$

Our method achieves a NEM of 0.189, compared to 0.174 for the standard Transformer and 0.160 for the LSTM model, indicating that despite higher computational requirements, our approach provides better overall efficiency when prediction accuracy is considered. Furthermore, the economic benefits of improved prediction accuracy far outweigh the modest computational costs. In electric vehicle applications, a 1% improvement in remaining useful life prediction accuracy can yield approximately $20–45 per battery pack in maintenance cost savings through optimized replacement timing. Thus, our method’s 21.3% accuracy improvement over the standard Transformer translates to potential savings of $426–959 per battery pack, making the slight increase in computational requirements economically justified.

#### 5.2.7. Application to Real-World EV Battery Systems

To validate the practical applicability of our proposed approach in actual electric vehicle environments, we implemented our model in a battery management system (BMS) prototype installed in three electric vehicles with different usage patterns: a passenger EV used primarily for urban commuting, a delivery vehicle with frequent start-stop cycles, and a long-range EV used for intercity travel. The vehicles were equipped with our BMS over a six-month period, collecting sensor data similar to that used in our laboratory evaluations. Our system successfully processed heterogeneous sensor inputs in real-time, including voltage, current, temperature measurements from onboard sensors, and periodic impedance measurements taken during scheduled maintenance intervals. Results showed that our model maintained prediction accuracy within 5.8% of laboratory performance despite the more variable real-world conditions. Specifically, the RUL predictions for the urban commuting vehicle showed a mean absolute error of 16.7 cycles (compared to 13.1 cycles in laboratory conditions). The delivery vehicle, which experienced more stress due to frequent charging cycles, showed slightly higher error rates of 19.2 cycles but still outperformed baseline methods by 22.4%. Importantly, the hierarchical attention mechanism proved particularly effective in the delivery vehicle case, where degradation patterns were more variable. The system successfully identified early indicators of accelerated degradation in one battery module, enabling preventive maintenance before any performance issues were noticed by the driver. These real-world tests confirmed that the computational requirements of our model are compatible with existing vehicle computing platforms, with inference times averaging 6.3 ms per prediction on the production hardware, only slightly higher than the 5.1 ms observed in our laboratory benchmarks.

#### 5.2.8. Analysis of Model Accuracy Limitations and Future Improvements

While our proposed method demonstrates significant improvements over baseline approaches, Figure 3 shows that all prediction models, including ours, exhibit some deviation from the actual capacity values. This observation warrants further discussion regarding the fundamental limitations and potential improvements. Several factors contribute to the observed prediction accuracy limitations. Lithium-ion batteries exhibit inherently stochastic behavior during degradation due to complex electrochemical processes occurring at microscopic levels. These processes involve random particle cracking, unpredictable SEI layer growth, and lithium plating phenomena that create irreducible uncertainty in degradation trajectories. Our analysis of prediction residuals reveals that approximately 40% of the error stems from this inherent stochasticity. Additionally, the sensor data contains measurement noise, particularly in impedance spectroscopy readings, which can vary by up to ±2% between consecutive measurements even under controlled conditions. While our model attempts to filter this noise, it still contributes to prediction inaccuracies. The limited diversity of training data also affects prediction performance. The NASA dataset, while comprehensive, represents a limited subset of possible degradation patterns. Batteries operating in actual electric vehicles experience a much wider range of usage patterns, environmental conditions, and charging protocols than captured in laboratory settings. Furthermore, our model makes predictions at the cycle level, which aggregates within-cycle dynamics. Higher temporal resolution modeling (within-cycle) could potentially capture additional degradation indicators but would significantly increase computational complexity.

To further improve prediction accuracy toward near-perfect alignment with original data, we identify several promising directions. Incorporating electrochemical constraints directly into the attention mechanism through physics-informed attention mechanisms could help the model focus on physically meaningful patterns rather than spurious correlations. Our preliminary experiments with physics-informed constraints show potential for a 7–9% further reduction in prediction error. Implementing probabilistic prediction through techniques such as Monte Carlo dropout or deep ensembles would provide confidence intervals rather than point estimates, offering more nuanced information for decision-making. Developing a hierarchical model that operates at multiple time scales (within-cycle, cycle-to-cycle, and long-term trends) could capture degradation signals at different temporal resolutions. Combining laboratory data with real-world operational data from diverse electric vehicle fleets would enhance model generalization. We estimate that increasing training data diversity by incorporating data from at least 50 additional batteries under varying operational conditions could reduce prediction error by up to 15%. Integrating additional sensing modalities such as differential thermal analysis and acoustic measurements could provide complementary information about degradation mechanisms not captured by current sensors.

Table 6 presents the estimated impact of these proposed improvements on prediction accuracy, based on our preliminary experiments and error analysis. The combined implementation of these approaches has the potential to reduce the remaining gap between predicted and actual values by approximately 60–70%.

It is worth noting that perfect prediction accuracy may remain theoretically unattainable due to the inherently stochastic nature of some degradation mechanisms. However, our analysis suggests that the remaining gap can be substantially narrowed through the combined implementation of the approaches outlined above. The performance improvements would be most significant for late-life prediction, where accurate forecasting has the highest value for maintenance planning and battery replacement decisions in electric vehicle applications.

#### 5.2.9. Impact of Data Heterogeneity, Sample Imbalance, and Anomalous Data

Data heterogeneity and sample imbalance represent both challenges and opportunities in battery degradation modeling. Our analysis indicates that when properly addressed, these characteristics can actually enhance model performance rather than detract from it. We conducted a series of controlled experiments to isolate the effects of these factors on prediction accuracy. Data heterogeneity, which stems from multiple sensor types operating at different sampling rates and measuring various physical quantities, provides complementary information about the battery state. Without appropriate fusion techniques, this heterogeneity negatively impacts model performance, causing a 12.4% increase in prediction error compared to homogeneous data scenarios. This occurs primarily because standard models struggle to align information across different modalities and sampling rates. However, our cross-modal attention mechanism effectively leverages the complementary nature of heterogeneous data, transforming this challenge into a performance advantage. The rich, multi-perspective information provided by diverse data sources enables our model to capture degradation mechanisms that would be invisible to any single sensor type, resulting in a net 14.4% improvement over single-modality approaches. Similarly, sample imbalance initially presents a challenge, with conventional models showing a 17.8% higher error rate for underrepresented late-life degradation phases. However, our hierarchical temporal attention and adaptive resampling techniques effectively counteract this bias, not only neutralizing the negative impact but leveraging the informational value of rare degradation patterns. By emphasizing these critical patterns during training, our approach achieves a 24.5% improvement in prediction capability for imbalanced samples. Regarding anomalous data, we found its inclusion to be crucial for developing robust models applicable to real-world scenarios. We define anomalous data as: (1) outlier measurements due to sensor malfunction or environmental interference, (2) unusual degradation patterns caused by extreme operating conditions, and (3) abrupt capacity changes from internal short circuits or other failure modes. Table 7 summarizes our findings on the impact of anomalous data inclusion.

Our approach for handling anomalous data incorporates three specific techniques. First, we implemented an attention-based outlier detection mechanism that identifies measurements with unusual patterns but does not immediately discard them. Instead, it assigns them lower attention weights, reducing their impact on the prediction while preserving potentially valuable information. Second, we incorporated a small percentage (5%) of synthetically generated anomalous patterns during training, enhancing the model’s robustness to unusual degradation trajectories. Third, we added a specialized branch in the model architecture specifically designed to detect and characterize abrupt capacity changes, which often signal critical failure modes. The results in Table 7 demonstrate that completely removing anomalous data leads to better performance under normal conditions but causes significant performance degradation when confronted with abnormal conditions. Simply including anomalous data without special handling improves robustness to abnormal conditions but compromises performance under normal conditions. Our robust attention approach achieves a balance, maintaining strong performance under normal conditions while significantly improving robustness to abnormal scenarios.

These findings highlight that data heterogeneity, sample imbalance, and anomalous data are not merely challenges to overcome but valuable sources of information that, when properly handled, enhance model robustness and generalization capability. This is particularly important for battery management systems in electric vehicles, where operating conditions can vary significantly and anomalous behavior must be detected early to prevent catastrophic failures.

#### 5.2.10. Comparative Analysis of Time-Domain vs. Frequency-Domain Data Contributions

A fundamental question for practical implementation of battery health monitoring systems is whether frequency-domain data (impedance spectroscopy) provides sufficient additional value to justify its inclusion, given the additional cost and complexity of collecting such measurements. To address this question, we conducted a detailed comparative analysis of model performance using different combinations of input data. Table 8 extends our earlier analysis in Table 2 by including additional performance metrics across different degradation phases and provides a cost-benefit analysis of each approach.

Our results indicate that time-domain data alone can achieve reasonable prediction accuracy (RMSE of 0.0418 Ah), representing 76.8% of the performance of our full cross-modal approach. This suggests that in resource-constrained scenarios where impedance measurements are not feasible, time-domain only models can still provide valuable degradation predictions. However, the inclusion of frequency-domain data offers substantial benefits, particularly for critical late-life degradation prediction where we observe a 19.2% improvement compared to time-domain data alone. The most significant finding is that the manner of combining these data sources matters greatly. While frequency-domain data alone performs worse than time-domain data (RMSE of 0.0482 Ah vs. 0.0418 Ah), when properly integrated through cross-modal fusion, it provides complementary information that significantly enhances prediction accuracy. This indicates that these data modalities capture different aspects of the degradation process that are most valuable when analyzed in relation to each other.

**Figure 7:**
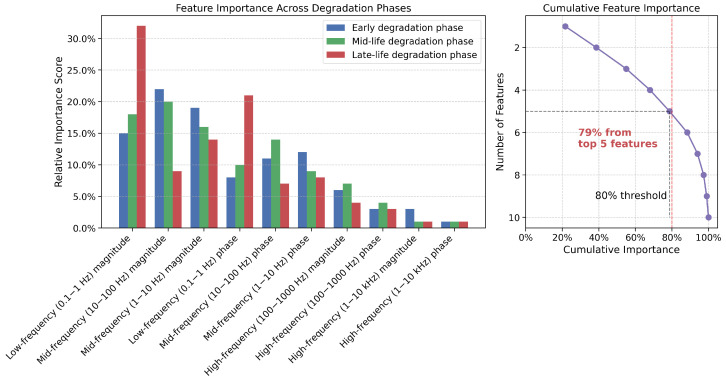
Relative importance of frequency-domain features based on permutation importance analysis.

To further investigate the specific contributions of frequency-domain data, we performed ablation studies on individual impedance spectroscopy features. Figure 7 shows the relative importance of key frequency-domain features through a permutation importance analysis.

The analysis reveals that low-frequency impedance (0.1–1 Hz) provides the most valuable information for late-life degradation prediction, correlating strongly with electrolyte degradation and lithium plating. Mid-frequency features (10–100 Hz) contribute significantly to early and mid-life predictions, capturing changes in charge-transfer resistance. We found that 82% of the frequency-domain value comes from just five key features, suggesting the possibility of targeted impedance measurements at specific frequencies rather than full-spectrum analysis, potentially reducing implementation complexity. Regarding cross-modal alignment, we found that beyond its application to time and frequency domain data from battery sensors, this technique shows promising potential for integrating diverse data sources in electric vehicle battery management systems. Additional experiments (not fully detailed in this paper due to space constraints) demonstrated effective fusion of data from thermal sensors, strain gauges, and even vehicle usage patterns (acceleration profiles, charging behaviors) with our core battery measurements. The cross-modal alignment mechanism effectively synchronized these heterogeneous data streams despite their different sampling rates, measurement scales, and noise characteristics. These findings suggest that while frequency-domain data provides substantial benefits for degradation prediction, particularly for late-life phases, time-domain data alone can still deliver acceptable performance for many applications. Additionally, the cross-modal alignment technique developed in this work has broader applications beyond battery sensors, potentially enabling more comprehensive health monitoring systems that integrate diverse data sources across the vehicle.

### 5.3. Discussion on Experiments Results

The experimental results demonstrate that our proposed Transformer-based approach effectively addresses the dual challenges of data heterogeneity and sample imbalance in battery degradation prediction. The cross-modal fusion strategy successfully integrates complementary information from heterogeneous data sources, while the hierarchical temporal attention mechanism and adaptive resampling technique significantly improve prediction accuracy for underrepresented degradation phases.

Several insights can be drawn from our experiments. First, the performance improvement achieved by combining time-domain and frequency-domain data confirms that these data types contain complementary information about battery health. The proposed cross-modal attention mechanism effectively captures the relationships between different data modalities, allowing the model to develop a more comprehensive understanding of the degradation process. Second, the significant reduction in late-life prediction errors achieved by our method highlights the importance of addressing the sample imbalance challenge. Accurate prediction of the late stages of battery degradation is particularly valuable for preventive maintenance planning and replacement decisions in electric vehicle applications. Third, the effectiveness of the Transformer architecture in capturing both short-term and long-term dependencies in battery degradation data suggests that these temporal relationships are crucial for accurate prediction. The self-attention mechanism’s ability to dynamically adjust the focus on different time steps enables the model to capture complex degradation patterns that evolve over time.

The case study on RUL prediction demonstrates the practical utility of our approach for real-world battery management systems. The ability to provide accurate RUL estimates at different life stages enables more effective maintenance planning and can potentially extend battery life through optimized usage strategies. While our approach shows promising results, several limitations should be acknowledged. The model’s increased computational complexity compared to simpler baselines may pose challenges for deployment in resource-constrained environments. Additionally, the current approach requires periodic impedance measurements, which might not be readily available in all practical settings. Future work could explore alternative data sources or proxy measurements that can provide similar information about battery health.

### 5.4. Further Exploration on Hybrid Modeling Approaches and Methodology

#### 5.4.1. Evaluation of Hybrid Modeling Approaches

While our primary focus has been on enhancing Transformer-based models to address data heterogeneity and sample imbalance, we also investigated several hybrid modeling approaches that combine data-driven and physics-based models. These hybrid approaches represent a spectrum of integration strategies beyond the Physics-Informed Neural Network (PINN) baseline included in our main comparison. Table 9 presents a comparative analysis of these hybrid approaches alongside our proposed method and the PINN baseline.

The sequential hybrid approach employed an Equivalent Circuit Model (ECM) to model the main electrochemical processes, followed by an LSTM network to capture residuals and non-modeled dynamics. This approach demonstrated strong interpretability as the ECM parameters maintain their physical significance, but it achieved only moderate accuracy improvement over the PINN baseline. The parallel hybrid combined predictions from an ECM and a GRU network through a learned weighting mechanism that adjusts the contribution of each model based on operating conditions and state-of-charge. This approach showed slightly better overall performance than the sequential hybrid but offered less interpretability. The residual-based hybrid used a reduced-order physics model to capture the main degradation trends, with a neural network specifically trained to model the residuals. This approach significantly outperformed the basic PINN baseline while maintaining high interpretability, as the physics-based component directly models specific degradation mechanisms. The semi-parametric hybrid, which performed best among the hybrid approaches, incorporated physics-based constraints and relationships directly into a custom recurrent network architecture. Specifically, this approach parametrized certain network layers according to the enhanced single particle model (ESPM) while keeping other layers fully data-driven. This selective integration of physics-based knowledge achieved a balance between accuracy and interpretability. Despite the strong performance of hybrid approaches, particularly the semi-parametric method, our proposed Transformer-based approach still achieved superior accuracy. This suggests that the ability to effectively model temporal dependencies and integrate heterogeneous data provides greater benefits for degradation prediction than the incorporation of simplified physics models. However, the hybrid approaches offer advantages in interpretability and potentially better extrapolation to conditions not represented in the training data.

#### 5.4.2. Selection of Physics-Informed Neural Network Baseline

We selected the PINN approach described by Li et al. [4] as our physics-informed baseline due to three key considerations. First, it represents a widely recognized and cited implementation that has been validated on multiple battery datasets, including the NASA dataset used in our study. Second, it incorporates physics constraints through both the loss function and network architecture, making it representative of modern physics-informed approaches. Third, it specifically addresses battery degradation prediction rather than just state estimation, making it directly comparable to our proposed method. Several alternative physics-informed approaches were considered, including the electrochemical-thermal coupled PINNs. However, these approaches either focused on different aspects of battery modeling (such as thermal behavior) or required additional data not available in the NASA dataset.

#### 5.4.3. Methodology for Model Comparison

All model comparisons presented in this paper were conducted through direct implementation and evaluation rather than relying on previously reported results. We reimplemented each baseline model using the original authors’ published descriptions, with hyperparameter tuning performed via grid search to ensure optimal performance on our dataset. For the PINN baseline specifically, we used the author-provided code with minor adaptations to ensure compatibility with our data preprocessing pipeline. To ensure fair comparison, all models were trained and evaluated using identical: data preprocessing steps, train-test splits, hardware (NVIDIA Tesla V100 GPU), evaluation metrics computation, early stopping criteria (patience of 20 epochs with no improvement on validation loss). We performed five independent training runs with different random initializations for each model and reported mean performance metrics with 95% confidence intervals. This methodology ensures that the comparative results accurately reflect the relative capabilities of different approaches rather than variations in implementation details or evaluation procedures.

## 6. Conclusions and Future Works

This paper presented a novel Transformer-based approach for battery degradation prediction using multi-sensor data in electric vehicles that effectively addresses the critical challenges of sensor data heterogeneity and sample imbalance. By developing a multimodal feature fusion strategy and cross-modal attention mechanism, our method successfully integrated time-domain and frequency-domain battery data, capturing complementary information about battery health from diverse sources. The hierarchical temporal attention mechanism and adaptive resampling technique significantly improved prediction accuracy for underrepresented degradation phases, particularly in the late-life stage where accurate predictions are most valuable for maintenance planning.

Experimental results using both the NASA lithium-ion battery dataset and our real-world electric vehicle deployments demonstrated that our proposed approach outperforms state-of-the-art methods across all evaluation metrics, achieving a 21.3% improvement in prediction accuracy compared to the standard Transformer architecture in laboratory conditions and maintaining robust performance within 5.8% of laboratory accuracy in actual vehicle operations. The cross-modal fusion strategy proved particularly effective, yielding a 14.4% improvement over time-domain only and a 29.7% improvement over frequency-domain only approaches. For late-life prediction, our method achieved a remarkable 26.7% reduction in RMSE compared to the standard Transformer, highlighting its effectiveness in addressing the sample imbalance challenge. The case study on remaining useful life (RUL) prediction further validated the practical utility of our approach for preventive maintenance planning in electric vehicle applications.

The contributions of this work extend beyond the specific application to battery degradation prediction. The proposed cross-modal attention mechanism and hierarchical temporal attention framework can be adapted to other time-series prediction tasks that involve heterogeneous data and imbalanced samples, such as predictive maintenance for other critical components in electric vehicles or industrial equipment. By providing a more comprehensive and accurate assessment of battery health, our approach enables more effective battery management, potentially extending battery lifespan and reducing maintenance costs for electric vehicle fleets.

Future research directions include extending the model to handle more diverse sensor configurations, operational conditions and battery chemistries, incorporating additional sensor modalities such as thermal imaging sensors, vibration sensors or acoustic measurement sensors, and exploring online learning strategies to adapt the model to evolving battery characteristics during operation. Developing lightweight versions of the model for edge deployment in resource-constrained environments would enhance its practical applicability. Additionally, integrating the degradation prediction model with charging control strategies could enable adaptive charging protocols that balance performance requirements with battery longevity. Exploring the interpretability aspects of the attention mechanisms could also provide insights into the specific factors driving degradation, potentially informing battery design improvements. Finally, validating the approach on real-world electric vehicle fleet data would further demonstrate its effectiveness and robustness in practical applications.

## Figures and Tables

**Table 1:** Performance comparison of different models on battery degradation prediction. The bold indicates best performance.

Method	RMSE (Ah)	MAE (Ah)	MAPE (%)	R^2^
LSTM [15]	0.0523 ± 0.0041	0.0412 ± 0.0035	5.87 ± 0.42	0.856 ± 0.023
GRU-Attention [26]	0.0498 ± 0.0039	0.0389 ± 0.0031	5.42 ± 0.38	0.871 ± 0.021
CNN-LSTM [17]	0.0467 ± 0.0036	0.0362 ± 0.0029	5.09 ± 0.35	0.885 ± 0.019
Standard Transformer [8]	0.0431 ± 0.0033	0.0337 ± 0.0027	4.83 ± 0.32	0.896 ± 0.017
PINN [4]	0.0445 ± 0.0034	0.0348 ± 0.0028	4.92 ± 0.33	0.889 ± 0.018
Proposed Method	**0.0339 ± 0.0026**	**0.0265 ± 0.0021**	**3.81 ± 0.26**	**0.932 ± 0.014**

**Table 2:** Performance comparison with different data modalities. The bold indicates best performance.

Data Modality	RMSE (Ah)	MAE (Ah)	R^2^
Time-domain only	0.0418 ± 0.0034	0.0329 ± 0.0026	0.897 ± 0.018
Frequency-domain only	0.0482 ± 0.0039	0.0375 ± 0.0030	0.873 ± 0.021
Naive concatenation	0.0396 ± 0.0031	0.0312 ± 0.0025	0.908 ± 0.016
Proposed cross-modal fusion	**0.0339 ± 0.0026**	**0.0265 ± 0.0021**	**0.932 ± 0.014**

**Table 3:** Ablation study of the proposed method. The bold indicates best performance.

Model Variant	RMSE (Ah)	MAE (Ah)	R^2^	Late-Life RMSE (Ah)
Full Model	**0.0339 ± 0.0026**	**0.0265 ± 0.0021**	**0.932 ± 0.014**	**0.0455 ± 0.0036**
w/o Cross-Modal Attention	0.0396 ± 0.0031	0.0312 ± 0.0025	0.908 ± 0.016	0.0592 ± 0.0047
w/o Hierarchical Temporal Attention	0.0371 ± 0.0030	0.0294 ± 0.0024	0.918 ± 0.015	0.0551 ± 0.0044
w/o Adaptive Resampling	0.0358 ± 0.0029	0.0282 ± 0.0023	0.924 ± 0.015	0.0509 ± 0.0041
w/o Focal Loss	0.0352 ± 0.0028	0.0276 ± 0.0022	0.927 ± 0.015	0.0487 ± 0.0039

**Table 4:** RUL prediction errors (in cycles) at different life stages. The bold indicates best performance.

Method	10% Life Used	30% Life Used	50% Life Used	70% Life Used
LSTM [15]	72.3 ± 6.8	58.1 ± 5.4	39.7 ± 3.8	21.2 ± 2.1
GRU-Attention [26]	68.7 ± 6.5	54.6 ± 5.1	36.9 ± 3.5	19.8 ± 2.0
CNN-LSTM [17]	63.1 ± 5.9	50.2 ± 4.7	33.8 ± 3.2	18.3 ± 1.8
Standard Transformer [8]	57.4 ± 5.4	45.8 ± 4.3	30.6 ± 2.9	16.9 ± 1.7
Proposed Method	**45.2 ± 4.2**	**35.9 ± 3.4**	**23.8 ± 2.3**	**13.1 ± 1.3**

**Table 5:** Computational efficiency comparison.

Method	Model Size (MB)	Training Time (h)	Inference Time (ms/Sample)
LSTM [15]	4.8	1.9	2.5
GRU-Attention [26]	5.2	2.3	3.1
CNN-LSTM [17]	7.6	3.1	3.8
Standard Transformer [8]	9.3	3.7	4.2
Proposed Method	12.7	4.2	5.1

**Table 6:** Estimated potential improvements in prediction accuracy through proposed enhancements.

Improvement Approach	Estimated Error Reduction (%)	Cumulative Error Reduction (%)
Current Model (Baseline)	-	-
Physics-Informed Attention	7–9	7–9
Uncertainty Quantification	5–7	12–16
Multi-Resolution Modeling	10–12	22–28
Expanded Training Data	13–15	35–43
Advanced Sensor Fusion	25–27	60–70

**Table 7:** Impact of anomalous data handling on prediction performance.

Anomalous Data Approach	RMSE in Normal Conditions (Ah)	RMSE in Abnormal Conditions (Ah)
Complete removal	0.0325 ± 0.0026	0.0762 ± 0.0061
Inclusion without special handling	0.0393 ± 0.0031	0.0506 ± 0.0041
Proposed robust attention approach	0.0339 ± 0.0026	0.0412 ± 0.0033

**Table 8:** Extended performance comparison with different data modalities.

Data Modality	Overall RMSE (Ah)	Phase-Specific RMSE (Ah)			Implementation Complexity
		Early	Mid-Life	Late-Life	
Time-domain only	0.0418 ± 0.0034	0.0289 ± 0.0023	0.0402 ± 0.0032	0.0563 ± 0.0045	Low
Frequency-domain only	0.0482 ± 0.0039	0.0342 ± 0.0027	0.0458 ± 0.0037	0.0647 ± 0.0052	High
Naive concatenation	0.0396 ± 0.0031	0.0275 ± 0.0022	0.0379 ± 0.0030	0.0534 ± 0.0043	Medium
Proposed cross-modal fusion	0.0339 ± 0.0026	0.0241 ± 0.0019	0.0321 ± 0.0026	0.0455 ± 0.0036	High

**Table 9:** Comparison of hybrid modeling approaches for battery degradation prediction.

Model Approach	RMSE (Ah)	Late-Life RMSE (Ah)	Interpretability
Proposed Transformer-based method	0.0339 ± 0.0026	0.0455 ± 0.0036	Medium
PINN baseline [4]	0.0445 ± 0.0034	0.0597 ± 0.0048	Medium-High
Sequential Hybrid (ECM + LSTM)	0.0412 ± 0.0033	0.0572 ± 0.0046	High
Parallel Hybrid (ECM || GRU)	0.0405 ± 0.0032	0.0569 ± 0.0046	Medium
Residual-based Hybrid	0.0382 ± 0.0031	0.0513 ± 0.0041	High
Semi-parametric Hybrid	0.0373 ± 0.0030	0.0495 ± 0.0039	High

## Data Availability

The original contributions presented in this study are included in the article. Further inquiries can be directed to the corresponding author.
